# Using self-organizing maps to develop ambient air quality classifications: a time series example

**DOI:** 10.1186/1476-069X-13-56

**Published:** 2014-07-03

**Authors:** John L Pearce, Lance A Waller, Howard H Chang, Mitch Klein, James A Mulholland, Jeremy A Sarnat, Stefanie E Sarnat, Matthew J Strickland, Paige E Tolbert

**Affiliations:** 1 Department of Biostatistics and Bioinformatics, Rollins School of Public Health, Emory University, Atlanta, GA, USA; 2 Department of Environmental Health, Rollins School of Public Health, Emory University, Atlanta, GA, USA; 3 School of Civil and Environmental Engineering, Georgia Institute of Technology, Atlanta, GA, USA

**Keywords:** Air pollution, Classification, Cluster analysis, Kohonen Map

## Abstract

**Background:**

Development of exposure metrics that capture features of the multipollutant environment are needed to investigate health effects of pollutant mixtures. This is a complex problem that requires development of new methodologies.

**Objective:**

Present a self-organizing map (SOM) framework for creating ambient air quality classifications that group days with similar multipollutant profiles.

**Methods:**

Eight years of day-level data from Atlanta, GA, for ten ambient air pollutants collected at a central monitor location were classified using SOM into a set of day types based on their day-level multipollutant profiles. We present strategies for using SOM to develop a multipollutant metric of air quality and compare results with more traditional techniques.

**Results:**

Our analysis found that 16 types of days reasonably describe the day-level multipollutant combinations that appear most frequently in our data. Multipollutant day types ranged from conditions when all pollutants measured low to days exhibiting relatively high concentrations for either primary or secondary pollutants or both. The temporal nature of class assignments indicated substantial heterogeneity in day type frequency distributions (~1%-14%), relatively short-term durations (<2 day persistence), and long-term and seasonal trends. Meteorological summaries revealed strong day type weather dependencies and pollutant concentration summaries provided interesting scenarios for further investigation. Comparison with traditional methods found SOM produced similar classifications with added insight regarding between-class relationships.

**Conclusion:**

We find SOM to be an attractive framework for developing ambient air quality classification because the approach eases interpretation of results by allowing users to visualize classifications on an organized map. The presented approach provides an appealing tool for developing multipollutant metrics of air quality that can be used to support multipollutant health studies.

## Background

The multipollutant approach to air pollution-related health research has a variety of objectives [1,2]; however, there is a common interest in the development of multipollutant exposure metrics that facilitate investigation of health effects associated with ambient air pollution mixtures [3]. This presents considerable challenges for health investigators, and several methodological strategies appear in the literature [1,3,4]. One prospective solution under active investigation is to use classifications or groupings as a means to characterize aspects of the multipollutant environment [5-8]. This is appealing to health investigators because classification of complex multipollutant data into specific categories can elucidate combinatorial patterns of interest and can be used to compare risk of an adverse health outcome observed within one air quality classification to that observed in another. Moreover, this is helpful statistically because classifications reduce the dimensionality of the data thus permitting one to assess effect sizes between classes rather than assessing effects associated with each potential combination of pollutant levels. Of course, an important consideration here is to how to define the classification groups, or in this setting, contrasts in multipollutant ambient environments [3,9].

Defining classifications are normally determined under two broad scenarios – when *a priori* grouping information is available and when it is not. For example, multipollutant combinations could be discriminated using prior knowledge of hypothesized biological pathways of effect [10] (e.g., inflammation) or known emissions sources (e.g., traffic) [11]. Alternatively, investigators without *a priori* information are turning to statistical methods that construct groupings by ‘learning’ from the data [5,7,8,12,13]. These approaches encompass a number of techniques that focus on the discovery of patterns and trends in data and can be categorized as being either ‘supervised’ or ‘unsupervised’ [14]. In supervised analyses the objective is to use an outcome measure in order to develop classification groupings that associate with or predict the outcome. With unsupervised approaches, there is no outcome measure and the objective is to identify groups in the data. This approach is often used to perform cluster analysis or data segmentation and thus groups are often referred to as clusters or modes. Once identified, groups are regarded as classes of observations which may provide potentially useful categories for further research. Such approaches show promise toward using classification for ambient air quality mixtures research; however, many challenges remain [1,3].

A starting point for a multipollutant characterization is to ask which combinations of pollutants are observed in the environment, how frequently they occur, and how long they persist. These issues are important because certain combinations may be more toxic than others. Therefore, such information could prove invaluable in addressing potential health effects and control strategies. The nature of unsupervised classification makes it well suited to address such questions; however, there are some concerns that results can be too general (i.e., classes are broadly defined) as most applications seek parsimonious solutions to the problem at hand [1,5]. Generally, a small number of groups is desired for simplicity of interpretation; however, health research presents a problem framework where describing ambient air quality with as much accuracy as possible is important for valid epidemiological studies. Therefore restricting health investigations to only a small number of scenarios has the potential for overlooking a rarer combination with strong impact on health [1]. Moreover, given the setting (e.g., multi-city analyses, hundreds of pollutants, sub-hourly measures, etc.), ambient air quality may not be well characterized by a few generalized scenarios. Such situations warrant exploration of techniques that are less governed by parsimony.

In this study, we present the self-organizing map (SOM) as a tool to create ambient air quality classifications because the method offers the benefit of a visual medium (the ‘map’) that can be useful for understanding classification results [15]. To illustrate, we apply SOM to eight years of day-level data from Atlanta, GA, for ten ambient air pollutants collected at a central monitor location in order to produce a variety of classes that represent subgroups of days with similar multipollutant profiles. Such classes can help identify potential pollutant combinations of interest and constitute a starting point for the development of scientific hypotheses and further study of health effects associated with ambient air quality mixtures.

## Methods

Our analytic aim is to formulate a discrete set of classes that represent high-density sub-regions in the multipollutant data space where days exhibit similar pollution patterns. In effect, this allows us to discover day-level multipollutant combinations that appear most frequently in our data. In this section we present our data, discuss data preparation, outline the self-organizing map algorithm, and describe our approach for applying SOM for developing multipollutant air quality metrics.

### Data

**Figure 1:**
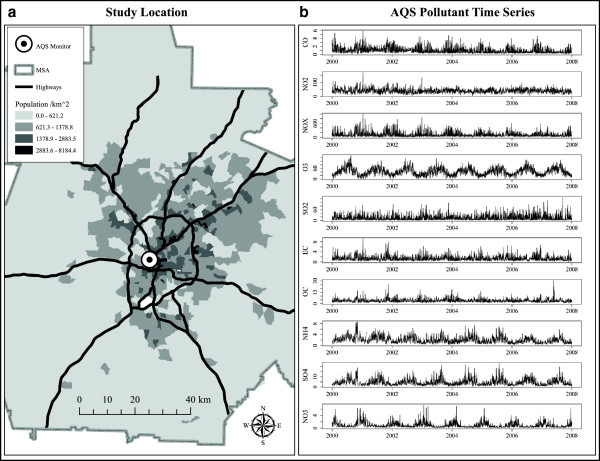
**Air pollution monitor data collected in Atlanta, GA, from 2000 to 2007.** Panel **(a)** Map of study region and air monitor location. Panel **(b)** Time series for pollutants collected at air monitor station.

Our data contain multipollutant time-series of daily concentration summaries for ten air pollutants sampled during the years 2000 to 2007 at a US EPA Air Quality System (AQS) monitoring station in Atlanta, GA (Figure 1). Temporal metrics chosen for this analysis followed National Ambient Air Quality Standards in an effort to identify multipollutant day types of potential health relevance. Pollutant included 1-hr maximum carbon monoxide (CO) in ppm, 1-hr maximum nitrogen dioxide (NO_2_) and nitrous oxides (NO*x*) in ppb, 8-hr maximum ozone (O_3_) in ppb, 1-hr maximum sulfur dioxide (SO_2_) in ppb, and five 24-hr average PM_2.5_ components in μg/m^3^: elemental carbon (EC), organic carbon (OC), nitrate (NO_3_), ammonium (NH_4_), and sulfate (SO_4_). This suite of ambient pollutants were chosen because measurements are fairly typical for many locations in the US and Western Europe. We note that these temporal metrics reflect a profile of day-level pollutant summaries for general air quality not simultaneous measurements of the air pollution mix at any single point in time during the day. See Table 1 for summary statistics.

**Table 1:** Summary Statistics of daily multipollutant data used to develop multipollutant day types in Atlanta during 2000-2007

**Variable**	**Mean**	**Standard deviation**	**Variance**	**Minimum**	**Maximum**	**Metric**
CO (ppm)	1.0	0.8	0.58	0.1	5.9	1-hr Max
NO_2_ (ppb)	40.3	15.5	239.18	4.4	172.0	1-hr Max
NO_*x*_ (ppb)	115.9	107.2	11498.48	5.0	996.0	1-hr Max
O_3_ (ppb)	41.8	20.4	424.94	2.0	132.1	8-hr Max
SO_2_ (ppb)	14.2	14.5	218.23	1.0	129.5	1-hr Max
PM_2.5_ EC (μg/m^3^)	1.4	0.9	0.93	0.1	9.3	24-hr Avg
PM_2.5_ OC (μg/m^3^)	4.0	2.3	5.25	0.4	30.9	24-hr-Avg
PM_2.5_ NH_4_ (μg/m^3^)	2.0	1.2	1.43	0.2	8.7	24-hr Avg
PM_2.5_ NO_3_ (μg/m^3^)	0.9	0.8	0.68	0.0	7.4	24-hr Avg
PM_2.5_ SO_4_ (μg/m^3^)	4.5	3.1	9.99	0.5	23.2	24-hr Avg

### The self-organizing map (SOM)

The SOM is an unsupervised method for multi-dimensional data reduction and display [15]. The goal of SOM is to compress information while preserving the topological relationships of the data. This is achieved by combining nonlinear projection and cluster methods to produce an ‘ordered vector quantization graph’. We find the approach to be a somewhat hybrid technique between multidimensional scaling (MDS) and *k*-means clustering as the result is a low-dimensional projection (the ‘map’) of class profiles (often called ‘clusters’, ‘prototypes’, or ‘nodes’ in SOM literature) that are arranged in a way that preserves the configuration of the original data space (the ‘organizing’) [14]. The resulting ‘map’, which is a projection of multidimensional space not geographic space, reveals interrelationships between classes and has proven beneficial in a variety of environmental settings [16,17]. The method has been shown to perform well in comparisons with traditional approaches [18,19]. To date, SOM applications in the field of air pollution have primarily focused on source apportionment with mixed success [20-22]; however, our objective here is more similar to clustering approaches to multipollutant environments [5,6,8] and thus our application of SOM is tailored accordingly.

#### SOM algorithm

**Figure 2:**
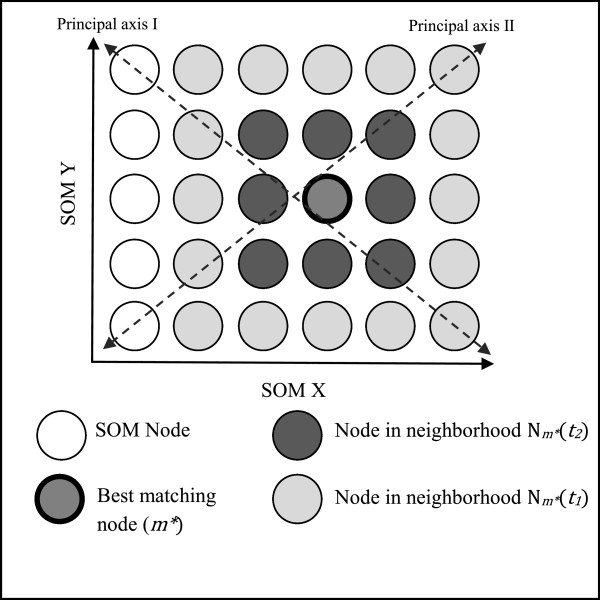
**A 6×5 self-organizing map (SOM) with rectangular topology.** Dashed lines highlight the principal axes (I and II), and dark grey tones identify activation of nodes in neighborhoods on the map for a training iteration *t*, with *t*_1_ < *t*_2_.

Applying SOM requires two components – the input data matrix and the output map (Figure 2). Here, the input matrix is our multipollutant data set, *Z*:

(1) $$Z=\left[\begin{array}{ccc} Z_{11} & \cdots & Z_{1p}\\ \vdots & \ddots & \vdots \\ Z_{n1} & \cdots & Z_{\mathrm{np}} \end{array}\right]$$

where *n* denotes the number of sampling days and *p* the number of pollutants. Each day is represented by a row *Z*_*i*_ within *Z*. The output collection of classes are displayed as nodes on the “map”, *M*:

(2) $$M=\left[\begin{array}{ccc} m_{1y} & \cdots & m_{\mathrm{XY}}\\ \vdots & ⋰ & \vdots \\ m_{11} & \cdots & m_{x1} \end{array}\right]$$

with each class *m* represented as a profile at location [*x*, *y*] on the map (Figure 1). Note *X × Y* determines the number of classes *k* and the arrangement (e.g., 1D or 2D) of *M*. The shape of *M* can be specified as either rectangular or hexagonal. Each *m* is characterized by a vector *w*_*m*_:

(3) $$w_{m}=\left[\mu_{m1},\mu_{m2},\ldots ,\mu_{\mathrm{mp}}\right]$$

where *μ* are ‘learned’ coefficients that define profile *m*.

Operationally, our SOM implements the following steps. First, given *M*, map initialization occurs with each *m* being assigned a preliminary *w*_*m*_ from a random selection of *Z*_*i*_’s. Then, sequential learning begins where, for each iteration *t*, the algorithm randomly chooses a day’s profile $Z_{i}^{\left(t\right)}$ from *Z* and then computes a measure of dissimilarity between the observation $Z_{i}^{\left(t\right)}$ and each $w_{m}^{\left(t\right)}$. Next, SOM provisionally assigns a best matching node m* (t) whose *w*_m*_ is most similar to each $Z_{i}^{\left(t\right)}$. Next, map development occurs via the *Kohonen learning process*:

(4) $$w_{m}^{\left(t+1\right)}=w_{m}^{\left(t\right)}+\alpha \left(t\right)N_{m*i}\left(t\right)\left[{\bar{Z}}^{\left(t\right)}-w_{m}^{\left(t\right)}\right]$$

where α is the learning rate, *N*_m*i_ is a neighborhood function that spatially constrains the neighborhood of m^*^ on *M*, and $\bar{Z}$ is the mean of pollutant values on days provisionally assigned to the nodes within the neighborhood set. The learning rate controls the magnitude of updating that occurs for *t*. The neighborhood function, which activates all nodes up to a certain distance on *M* from m^*^, forces similarity between neighboring classes on *M*. Eq. (4) updates coefficients within a neighborhood of m^*^, where the impact of the neighborhood decreases over iterations.

SOM results are dependent on both *α* and *N* and thus mappings are sensitive to these parameters^30^. We note that for classification *α* should start as a small number and be specified to decrease monotonically (e.g., 0.05 to 0.01) as iterations increase [23]. Similarly, the range of *N* should start large (e.g., 2/3 map size) and decreases to 1.0 over a predetermined termination period (e.g., 1/3 of iterations), which allows fine adjustment of the classes to occur.

Training continues for the number of user-defined iterations. Kohonen recommends the number of steps be at least 500 times the number of nodes on the map. Once training is complete, results include final coefficient values for each class profile *w*_*m*_, classification assignments for each day *Z*_*i*_, and coordinates for class nodes on *M*. The final step is to visualize the class profiles by plotting the map. For additional details please refer to the SOM book by Kohonen [15].

### Applying SOM for multipollutant air quality characterization

In this section we describe our approach for applying SOM to generate a classification of multipollutant day types. However, before we can apply SOM we need to perform three preliminary steps: 1) standardize the data, 2) define dissimilarity, and 3) choose how many classes to look for in the data. Decisions made for each of these steps can have consequences on results; however, there is typically not a single right answer and decisions are usually guided by subject matter knowledge or data-based heuristics [14].

#### Training set selection and data standardization

A preliminary step needed to prepare the data for unsupervised classification is to identify an appropriate training data set. Here this is achieved by identifying days in which we have complete observations for all pollutants. This results in a training data set composed of 2724 days. Another important preliminary step to unsupervised classification is to standardize the variables, particularly those measured in different units, to have a mean zero and standard deviation of one before analysis [14]. To achieve this we standardize by subtracting mean values and dividing by standard deviations as this removes the absolute differences between variable magnitudes but retains the ratios between variable amplitudes.

#### Defining similarity of days

The definition of dissimilarity is essential to any unsupervised classification approach [14]. In this study, we define using the Euclidean distance (*D*) between observations in multidimensional data space. With our data, *D* is calculated as:

$$D\left(Z_{i},Z_{i*}\right)={\left[\sum_{j=1}^{p}{\left(Z_{\mathrm{ij}}-Z_{i*j}\right)}^{2}\right]}^{1/2}$$

where (*Z*_*i*_*, Z*_i*_) represents a pair of observations for the *i*th and i*th days, Z_*ij*_ and *Z*_i*j_ are the *i*th and i*th values of pollutant *j*, and *p* is the total number of pollutants.

#### Selecting map size

The choice for map size, analogous to selecting the number of classes *k*, typically depends on the goal of the analysis. Here we are interested in creating a set of specific classes that can be used as a multipollutant metric of ambient air quality and thus we present an approach to framing the problem towards our objective that involves common strategies taken in cluster analysis and data segmentation.

First, we graphically explore the grouping structure of our data by applying multi-dimensional scaling (MDS) in order to produce a two-dimensional representation of the data that preserves the pairwise distances between observations as well as possible [14]. MDS representations of high-dimensional data are often useful in unsupervised classification because they provide visual means of exploring the data that can be useful for identifying boundaries in data sets that exhibit grouping structure.

Second, we use a data-based method to identify the number of classes *k* by evaluating the ratio between the within-class variance and the between-class variance as a function of the number of classes. The expectation here is that if there is really k* groupings in the data then the right class solution will present itself with a substantial shift (i.e., elbowing) in the evaluation statistic. To investigate this variance ratio, we use the Calinski-Harabasz Index (*CH*) [24], which is defined as:

$$\mathrm{CH}=\frac{SS_{B}/\left(k-1\right)}{SS_{W}/\left(n-k\right)}$$

where *SS*_*B*_ is the overall between-class sum of squared deviations, *SS*_*W*_ is the overall within-class sum of squared deviations, *k* is the number of classes, and *n* is the number of observations. Well-defined groupings have a large between-class variance and a small within-class variance and thus the larger the *CH* index, the better the grouping structure of the partition. We chose the *CH* index because it is well suited for grouping solutions based on Euclidean distance and it performed well in a comparison of several statistics focused on identifying groups in multivariate data [24].

Finally, we are interested in providing a solution that produces a reasonable, dimension reduced, approximation of the original data. To explore this aspect, we estimated the percentage of variance explained by each class solution by fitting a regression model to predict each pollutant using a categorical variable for each classification solution as the predictor. We summarize results using the adjusted R^2^, where an increasing R^2^ indicates an increasing ability of a class solution to approximate the original data.

Ultimately, we would like classifications to provide meaningful categories of ambient air quality for epidemiological research. Given this, we restrict the potential range of solutions using a minimum of 2 classes up to a maximum of 30 classes as potential sample size is a concern. Of course, the definition of meaningful is likely to be hypothesis specific and thus we note that our strategy may be adapted to suit other scenarios.

#### SOM implementation

Implementation of the SOM algorithm in this study was performed using the ‘kohonen’ package in the R environment for statistical computing [23,25]. For each map size, ten random initializations were chosen and, once initialized, training of the SOM was accomplished by setting the algorithm to run a number of iterations equal to *k* classes × 500 for each size. The learning rate α and the neighborhood function *N* were specified α to decrease linearly from 0.05 to 0.01 and set *N* to start with a value that covered 2/3 of all node-to-node distances, decrease linearly, and terminate after 1/3 of the iterations had passed. A random initialization scheme yielding the most frequent quantization error (QE) was used for evaluation. QE, a standard output from the software, is the weighted average of Euclidean distances between the input days and the class profile to which they are assigned. For more detail on implementation of SOM in R please refer to Wehrens and Buydens [25].

#### An example map

To demonstrate SOM we developed a ‘medium’ size map of multipollutant day types representing the range of day-level multipollutant combinations observed in our data. We visualized class profiles on the map using pie segment diagrams where the average daily concentration of a pollutant under a given profile is indicated by size. The temporal frequencies and durations were calculated for each day type. In addition, we use the SOM grid to present summaries of long-term trends, seasonality, meteorology, and pollutant concentrations within each class as such summaries are anticipated to be useful for air pollution epidemiologic investigations.

We surveyed the following quality indicators: class reliability, spatial organization, and map distortion. Statistically speaking, a reliable class is a grouping of days with low dispersion. To assess, we use a coefficient of variation (CV), which was calculated as the ratio of the standard error (standard deviation / $\sqrt{n}$) of the mean to the mean dissimilarity of days within each class, times 100. The spatial organization was assessed by comparing the location of class profiles on the SOM to a dendrogram created by applying Ward’s hierarchical clustering to the SOM class profiles [26]. Map distortion occurs because SOM is restricted to mapping over a finite grid and therefore the actual strengths of dissimilarity may vary between adjacent class profiles in different regions on the map. To better visualize these divergences or ‘distortions’ between class profiles on the map we applied an additional visualization technique known as Sammon Mapping [14]. Finally, we compared results with applications of *k*-means and Ward’s clustering algorithms [5].

## Results

### SOM mapping of multipollutant day types

#### Map size

**Figure 3:**
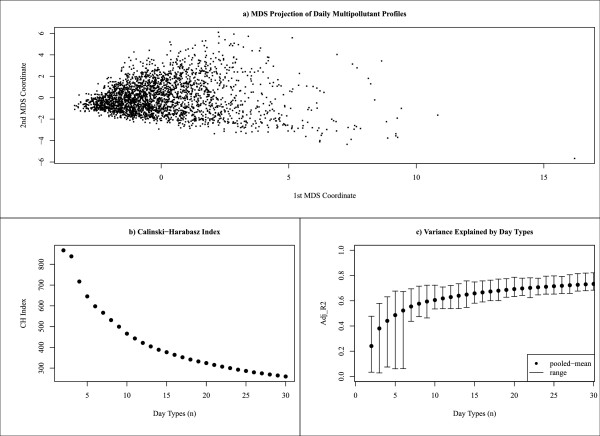
**Measures used to aid selection of number of day types for SOM classification system.** Panel **(a)** two-dimensional representation of multipollutant data created using multidimensional scaling. Panel **(b)** Calinski-Harabasz clustering index values for each class number tested. (Higher values are better.) Panel **(c)** resulting mean (± range) adjusted R^2^ values from regression models fit to each pollutant using the SOM classification as the predictor for each class number.

The MDS display indicates that our data are not composed of distinct well-separated groupings, as no clear boundaries between sets of observations are obvious (Figure 3a). On the left side we see a compressed region of relatively ‘clean’ air days and moving to the right we see an expansion of days, which illustrates the increasing dissimilarity of days driven by secondary pollutants (e.g., O_3_, SO_4_) and days driven by primary pollutants (e.g., CO, NO_2_). A graphical display of the CH statistic (Figure 3b) identified a two-class system as the best ‘clustering’ for our dataset with substantial drops occurring after *k* equals three classes. Inspection revealed that a two-class solution generalizes air quality as either days when all pollutants were high or days when all pollutants were low. The three-class solution described days as conditions when either all secondary pollutants were high, all primary pollutants were high, or all pollutants were low. Plotting the pooled adjusted R^2^ for each map size indicated a positive nonlinear relationship between class number and the map’s ability to reflect the overall variability in the daily pollutant measures (Figure 3c). However, inspection of the ranges reveals that notable improvements occur at *k* = 7, 10, 15, 19, and 28 partitions. This trend reflects the capturing of more subtle features in the data as *k* increases.

These findings reflect the challenge of determining *k* for our data because they do not exhibit groupings with clear boundaries. This is an important finding because traditional cluster statistics are known to perform poorly when data are not well separated [27]. Given this, and the fact that CH identified very generalized classifications, we focus our attention on the R^2^ statistic as it seems to do quite well in capturing more subtle features in our data. Here, we see that the best mapping of our data occurs at *k* = 28; however, this would produce groupings with expected class sample sizes of less than one hundred days per class, a situation likely to result in lower statistical power. Therefore, we choose to illustrate the approach with *k* = 16 as this number of classes suggests a reasonable mapping of our data given the sufficient balance between variance explained (mean R^2^ = 0.67) and expected class sample size (n = 170 days). Additionally, selecting 16 classes allows construction of a 4 × 4 two-dimensional map (i.e., a 4 × 4 SOM), a low-dimensionality that facilitates preservation of topological structure and improves visualization of interrelationships between classes [15].

#### The example map

**Figure 4:**
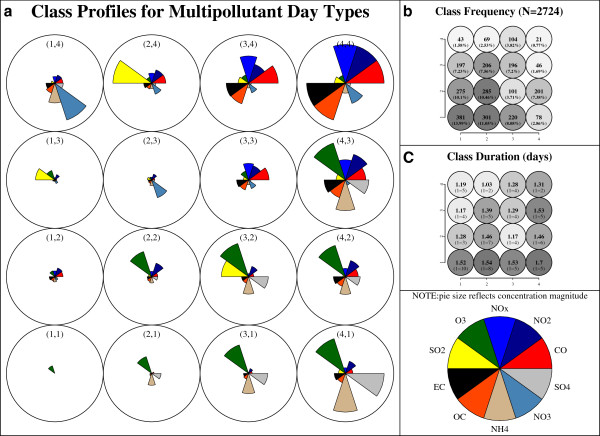
**A 16-class representation of multipollutant air quality days observed in Atlanta, GA, during the years 2000 to 2007 identified with SOM analysis.** Panel **(a)** discovered class profiles describing each multipollutant day type. Pollutant concentrations under each class are represented by the size of the pie and coordinate indices are in parentheses. Panel **(b)** resulting frequency counts for number of air quality days classified under each day type. Percentages are listed in parentheses. Panel **(c)** mean (range) number of days in which a day type persisted. (Lighter grey tones indicate lower values and darker shades indicate higher values).

**Figure 5:**
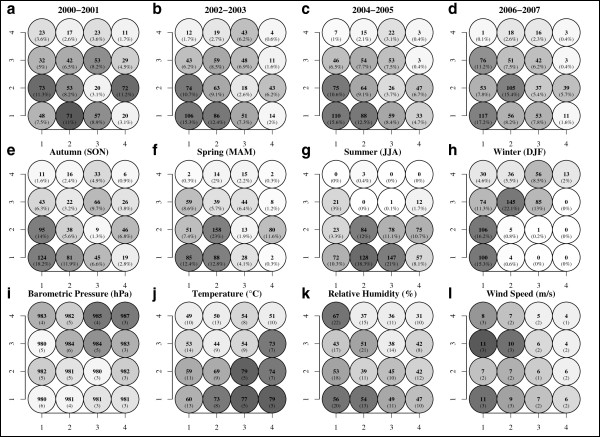
**Bi-annual frequencies, seasonal frequencies, and meteorological summaries for each SOM multipollutant day type.** Panels **(a-d)** map bi-annual frequency counts for each multipollutant day type to the SOM grid. Panels **(e-h)** map seasonal frequencies of day types to the SOM grid. Panels **(i-l)** map the average (standard deviations) local weather conditions observed under each day type to the SOM grid. (Lighter grey tones indicate lower values and darker shades indicate higher values).

Application of a 4 × 4 SOM identified classes that define a broad range of multipollutant day types (Figure 4). Globally, we see that the principal axes along the diagonals of the map reveal two primary modes of variation in our data: days dominated by primary pollutants such as CO, NO_2_, EC, and OC (bottom left to upper right), and days dominated by secondary pollutants such as O_3_, SO_4_, and NH_4_ (top left to bottom right). Zooming in, we see the bottom left corner of the map represents types of days that occurred frequently and exhibited relatively low pollution conditions. Consistent with these relatively clean atmosphere day types are the low pressure, high wind speed, and high relative humidity conditions associated with strong atmospheric mixing and rain (Figure 5). In the upper right, types represent rather infrequent days dominated by higher primary pollution (Figure 4). The pollutant combinations dominating these poorer air quality days suggest that pollution released at ground level (mobile sources) was concentrated due to poorer mixing meteorological conditions and potential inversions (i.e., high barometric pressure, low wind speed, and low humidity) (Figure 5).

The upper left identifies types dominated by high ammonium nitrate (type [1,4]) that occurred primarily in winter (Figure 4) and days with relatively increased SO_2_. The bottom right consists of days dominated by high ammonium sulfate and ozone (type [4,1]) that occurred in summer (Figure 4). Moving up from the bottom right, type [4,2] represents days that appeared to be more acidic, containing increased SO_2_ and less neutralized sulfate (i.e., more ammonium bisulfate). In the center of the map, we see that types reflect more moderate, less distinguishable air quality days. It is important to note that evaluation of similar map sizes indicated that the same primary modes of variation were revealed at each level of generalization. Specifically, a 3 × 3, 4 × 3, 5 × 4 and 6 × 5 SOM reveal the same broad patterns of multipollutant day types, with days dominated by primary pollution in one corner and days dominated by secondary pollution in the other. However, as map size increased distinctions between corners of the SOM became more apparent and less dominant day types (particularly involving SO_2_) were revealed. Finally, we found that SOM multipollutant day types, while certainly impacted by source emissions, were generally more dependent on meteorology than on expected source groupings [28].

The organization of multipollutant profiles on the SOM reveals through proximity that relatively clean days (type [1,1]) are most different from the days dominated by high primary pollutants (type [4,4]) and that the highest ozone-ammonium sulfate days (type [4,1]) are most different from the highest ammonium nitrate days (type [1,4]). Moreover, lower secondary days (type [2,1]) are more similar to moderate secondary days (type [3,1]) and lower primary days are more similar to moderate primary days. This organization lays a qualitative framework that can be used to begin understanding relationships between multipollutant day types and can provide insight into potential air quality contrasts. However, we reiterate that the relative magnitude of the interclass dissimilarity does vary across the map and thus additional techniques (e.g., Prim’s Minimal Spanning Tree [22]) may prove useful in further understanding class interrelationships.

The temporality of day types indicated heterogeneous frequencies of the kinds of multipollutant days experienced (Figure 4b) and moderately variable persistence of such types (Figure 4c). The least frequent day types were associated with the highest pollution levels and the most frequent were associated with moderate to relatively low pollution levels. The average duration for our collection of multipollutant day types was less than two days with average ranges being around one to five days. Types with greater persistence were dominated by relatively high secondary pollution or relatively low pollution, and shorter duration day types were dominated by primary pollutant combinations or single-pollutant episodes (e.g., elevated SO_2_ days – type [2,4]). It is important to note that the relatively specific nature of our classification captures transitions in air quality that are rather subtle in nature (e.g., transition from a moderate secondary day to a high secondary day). In contrast, a more general classification (e.g., two-class system) would be expected to result in longer durations and the transitions in air quality would be less subtle.

**Figure 6:**
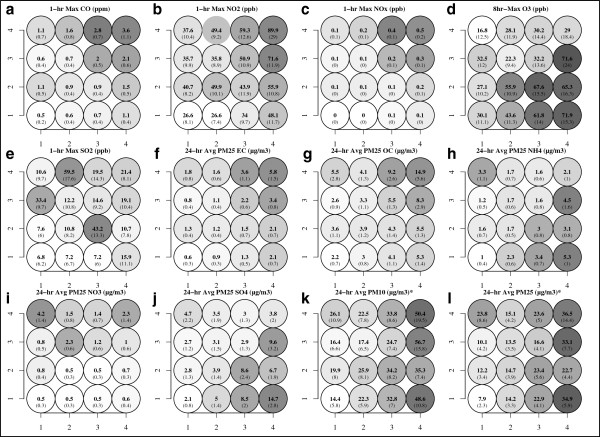
**Single pollutant summaries for each SOM multipollutant day type.** Panels **(a-l)** map the observed pollutant means (standard deviation) on days assigned to each class. *Pollutants not included SOM training analysis. (Lighter grey tones indicate lower values and darker shades indicate higher values).

The biannual and seasonal frequencies of each day type illustrate a strong association with long-term trends and seasonality (Figure 5). Biannual frequencies indicate a decrease in day types dominated by relatively high primary pollution and a steady persistence of secondary day types. As expected, the distribution of seasonal frequencies on the SOM was consistent with expected air quality across seasons (e.g., summer dominated by secondary pollutants and winter dominated by primary pollutants). Examining pollutant concentrations under each day type (Figure 6) highlights potential contrasts of interest for subsequent testing. For example, comparisons of similar O_3_ concentrations (Figure 6d) with differing co-pollution (e.g., type [3,2] and [4,2]) or similar PM_2.5_ (Figure 6l) with varying co-pollutants (e.g., type [1,4] and [3,4]) could be of interest in a health investigation.

**Figure 7:**
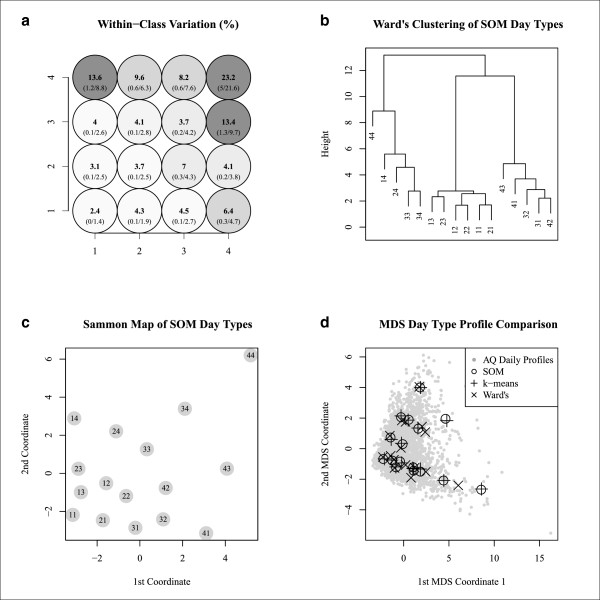
**Evaluation measures used to characterize 4 × 4 SOM quality.** Panel **(a)** presents the coefficient of variation for within-class similarity in bold and the standard error/mean in parentheses. Panel **(b)** presents a dendrogram of SOM classes labeled with *xy* coordinates. Panel **(c)** presents a Sammon map of SOM classes labeled with *xy* coordinates. Panel **(d)** presents a multidimensional scaling projection of daily multipollutant profiles and class profiles generated from SOM, *k*-means, and Ward’s clustering approaches.

Mapping the CV for within-class dissimilarity across the SOM provides understanding of within-class dispersion that could play an import role in the inferential confidence associated with each multipollutant day type (Figure 7a). Here, a lower value is better and thus we see that the higher pollution day types in the upper right corner have the greatest within-class dispersion and that the lower pollution day types in the bottom left have the most uniformity (Figure 7a). Ward’s clustering of class profiles confirms that SOM arranged day types so that similar types are neighbors (Figure 7b). Sammon mapping allows us to see that the magnitude of dissimilarities between day types varies across the map (Figure 7c). This projection of classes can help refine understanding of inter-class relationships between day types. Finally, comparison of SOM with *k*-means and Ward’s indicates similar day types were derived from the three techniques (Figure 7d). Class assignments across techniques found direct agreement for 94% of days between SOM and *k*-means, 55% of days between SOM and Ward’s, and 54% of days between *k*-means and Ward’s. These results agree well with comparisons in the literature [18,19] and establish that SOM can classify multipollutant day types in a manner that is comparable with more conventional methods.

## Discussion

Our application of SOM provides an unsupervised approach to producing classification systems for summarizing ambient air quality as a collection of day types. The classes presented distinguish contrasts in the ambient air quality environment through the formation of categories that generally reflect high frequency modes of the multipollutant distribution. In effect, this partitioning of the multipollutant ambient environment results in classes that have the characteristic of being numerically optimized into a collection of what many describe as ‘natural groupings’ in the data [14]. Such classes can play an important role in air pollution mixtures research as we have demonstrated their usefulness in identifying the types of multipollutant combinations that occur, their temporality, and for summarizing external variables of interest (Figures 4, 5 and 6).

An important point of discussion is if there is added value of using SOM versus more standard unsupervised approaches for developing multipollutant metrics of ambient air quality. Our comparison of SOM with cluster analysis identified that classifications agreed strongly with *k*-means, and to a lesser extent Ward’s solutions (Figure 7d); however, our rationale for introducing SOM is not that we expect to find different groupings than cluster analysis would provide (in fact we hope for agreement) but rather that we anticipate need to produce complex classifications for mixtures research that may be difficult to understand using traditional approaches. It is in these situations that application of SOM will likely be of greatest value as the additional visualization provided by the map facilitates understanding of interrelationships among classes. This benefit can be useful in settings requiring larger numbers of classes and aid in scenarios when classes need to be compared. Beyond the map, we note that SOM offers many additional benefits such as the ability to project classifications onto new data, classification of data with missing values, and extensions that facilitate supervised learning [23].

Of course, SOM is not without limitations. One shortcoming is the restriction of the map to a finite grid, as this constraint limits potential of the map to provide precise information about class dissimilarity. Another drawback is the need for the underlying map grid to be developed using sets of numbers (e.g., 3 × 3, 4 × 3, etc.) that generalize to shapes such as a square or rectangle. On the other hand, if a non-figurate solution is desired a 1-dimensional grid can be developed; however, this configuration is less appealing for visual interpretation of complex classifications as topological structure is more difficult to preserve. Nevertheless, the additional information provided by the map, as well as the flexibility of the technique, favors adding the SOM to the analytic toolkit for ambient air quality mixtures research.

Another important point of discussion is the broader challenge of determining the ‘right’ number of classes for a specific unsupervised analysis. This remains an open problem because there is no direct measure of success and thus it can be difficult to ascertain the validity of inferences drawn from an unsupervised learning algorithm [14]. As such, a reliance on heuristic arguments for judgments as to the quality of results has resulted in a complex situation that has led to many proposed solutions [24]. To simplify, there are two kinds of errors that can occur when making this decision – selecting a solution with either too few classes or selecting a solution with too many classes. The consequences depend on the context of the problem; however, here we suggest that selecting too few classes might be considered more serious because it could lead to important information being lost by merging important distinctions under highly generalized classes. On the other hand, defining “too many” classes can result in classes that are too similar or present small within-class sample sizes leading to reduced statistical power. With SOM, a choice of “too many” classes will simply subdivide a larger class into neighboring classes, an outcome we argue as less severe for our application than over generalization.In this study, we expand from a traditional clustering approach in that we wanted to identify a variety of classes that well describe the multipollutant features in our data with a relaxed emphasis on parsimony. Moreover, we also wanted to retain potential applicability of categories for epidemiological investigation. To achieve this goal, we presented an approach for identifying a ‘reasonable’ number of classes that emphasized aspects of classification representativeness and considered potential statistical power (Figure 3). This strategy found a 16-class system as an attractive solution for this data as it provided a balance between variance explained and potential sample size. However, we note that this should not be interpreted as the ‘right’ solution for this data as any partition of the data has to potential to produce interesting results.

Another broadly related issue is the likely influence of variable measurement error among the pollutants on the ability to validate a given class solution. Research has shown that increasing measurement error impedes the ability of unsupervised methods to find the ‘right’ grouping structure in the data [29]. Strategies for dealing with this issue, such as variable weighting, have promise; however, given that measurement error is expected to be a major difficulty in multipollutant air quality characterization [1,3], more research is needed to develop mixtures relevant tactics. Finally, health investigators should be aware of the potential for conclusions based on data grouping – particularly subsequent health investigations that utilize groupings – to be sensitive to different aggregations of the same multipollutant data. Given the potential implications of this particular issue, we suggest analyses consider testing multiple classification solutions before drawing conclusions.

The development of multipollutant metrics of ambient air quality for investigating population-level health impacts is an open problem and thus there are many opportunities for future research. A natural extension of the work presented herein is to investigate the health risks associated with multipollutant categories of ambient air quality. Additionally, profiles could be used to aid joint effects studies. Other areas of research that need to be explored are multipollutant spatial and spatiotemporal classifications. Finally, we note there is still much room for improvement when applying classification to create multipollutant classes of ambient air quality. In particular, further exploration of class number selection strategies, dissimilarity metrics, high-dimensional issues, and standardization approaches could benefit air pollution mixtures research.

## Conclusion

We find SOM to be an attractive framework for classifying day types for ambient air quality characterization because the approach produces classifications equivalent to traditional techniques with the benefit of a map that provides an organized visualization of class profiles. This additional feature of SOM promotes understanding of potentially complex interclass relationships that could prove useful in multipollutant research settings requiring larger classification systems.

## Abbreviations

SOM: Self-organizing map; MDS: Multi-dimensional scaling; CH: Calinski-Harabasz index.

## Competing interests

The authors declare no financial competing interests.

## Authors’ contributions

JP introduced the idea of self-organizing maps for multipollutant research, performed all analyses, and drafted the manuscript. LW participated in the conceptual approach, describing the algorithm, and revising the manuscript. SS participated in the conceptual approach and revisions of the manuscript. HC participated in the description of the algorithm. MK participated in the conceptual approach. JM provided the air quality descriptions. JS participated in the conceptual approach and revisions of the manuscript. MS participated in the conceptual approach and revisions of the manuscript. PT participated in the conceptual approach and revisions of the manuscript. All authors read and approved the final manuscript.
